# Color image encryption based on chaotic compressed sensing and two-dimensional fractional Fourier transform

**DOI:** 10.1038/s41598-020-75562-z

**Published:** 2020-10-29

**Authors:** Xingyuan Wang, Yining Su

**Affiliations:** School of Information Science and Technology, Dalian Maritime University, Dalian, 116026 China

**Keywords:** Computer science, Information technology

## Abstract

Combining the advantages of structured random measurement matrix and chaotic structure, this paper introduces a color image encryption algorithm based on a structural chaotic measurement matrix and random phase mask. The Chebyshev chaotic sequence is used in the algorithm to generate the flip permutation matrix, the sampling subset and the chaotic cyclic matrix for constructing the structure perceptual matrix and the random phase mask. The original image is compressed and encrypted simultaneously by compressed sensing, and re-encrypted by two-dimensional fractional Fourier transform. Simulation experiments show the effectiveness and reliability of the algorithm.

## Introduction

Images can truly show us the real world, especially color images are becoming more and more important in life. For a color image, it has three elements of R, G, and B, so it contains more information than grayscale images, its original features, large amount of data, high redundancy, high correlation between pixels. In order to protect image information, major contributions have been made in the fields of steganography^1,2^, and encryption^3–5^. In recent years, chaos has some ideal cryptographic characteristics such as initial value sensitivity and pseudo-randomness, which makes the chaotic encryption scheme widely used^6–17^. For example, Ahmad et al. proposed a chaos-based high-key image encryption scheme that makes even one round of encryption, the key space is very large^9^. For the small number of keys and simple key transmission method, Wu et al. used 4D cat mapping and elliptic curve ElGamal for asymmetric encryption^10^. Considering the increase in image size, Chai et al. proposed to divide the image into blocks for scrambling and diffusion^11^.

Donoho proposed a new sampling reconstruction technology, this technology is called compressed sensing^18^. Various CS encryption schemes with high efficiency and low data volume have been proposed, but CS-based encryption schemes are not resistant to selective plaintext attacks^19^. Since the measurement matrix satisfies low cross-correlation, a random matrix such as a Gaussian random matrix has a large-capacity memory and high complexity^20,21^. Therefore, in order to eliminate the shortcomings of random matrix, an encryption scheme based on CS and chaotic system^22–25^ is designed, and deterministic matrix is introduced instead of random matrix^26,27^. For example, Naidu proposes to use Euler lattice to construct a binary perceptual matrix, but this is limited to medical image and greatly limits the scope of application^28^. Combining the advantages of chaos, this paper proposes a structural chaotic matrix, using Chebyshev map to construct a flip scrambling matrix, a chaotic-based cyclic matrix, and a sampling subset. These three parts are completely determined structures. Although CS is used to reduce the amount of data re-encryption, the characteristics of color images still exist.

Due to the high speed and parallel processing of optical images, a large number of optical-based image encryption schemes have been proposed^29–31^. Although classical optical encryption based on double random phase mask is easy to attack^32^, it lays a foundation for subsequent optical encryption schemes. Use FrFT, Fresnel transform to enhance security^33,34^ to overcome various attacks. For example, Farah et al. proposed a new method for encrypting optical images using fractional Fourier transform, DNA sequence manipulation, and chaos theory. The encryption method has high security but high complexity and cost^35^. In order to avoid high complexity and too large data to transmit, a combination of CS and optical encryption is proposed^36–40^. Zhang et al. proposed a fast and effective color image encryption scheme based on two-dimensional compressed sensing and fractional Fourier transform^36^. To solve the risk of linear transformation in image encryption technology, Zhou et al. proposed an image encryption scheme that combines compressed sensing and nonlinear fractional Merlin transformation^37^. In order to reduce the amount of data, the algorithm uses Chebyshev map to generate chaotic sequences to construct a deterministic structured sensing matrix and a random mask. The image is first compressed and subjected to two-dimensional fractional Fourier transform. Through simulation experiments, the algorithm has good security performance and can resist common attacks.

To overcome the above difficulties, we propose a structure-sensing matrix and two-phase random mask color image encryption algorithm. The main contributions of this paper are: (1) Combining the advantages of a structured random perception matrix and a chaotic structure, a random perception matrix with a secure structure is proposed. The novelty of the matrix is that the original signal is flipped, the flipped coefficient is measured quickly and pseudo-randomly, and the final sample is obtained by sampling. (2) In real life, the utilization rate of color images is higher, but the color image data has a large amount of data, high redundancy, and high correlation between pixels. Our proposed encryption scheme can overcome these difficulties. Using CS can simultaneously compress and Encryption, reducing data volume and degrading transmission costs. (3) The proposed encryption scheme overcomes the difficulty that the previous Fourier transform-based encryption schemes are easily attacked. The two-dimensional fractional Fourier transform is used to increase the key space. The experimental results and security analysis show the security of the algorithm.

## Algorithm foundation

### Compressed sensing

Compressed sensing theory is a brand new signal sampling compression. Suppose that the signal $f$ of size $N \times {1}$ can be expressed as sparse basis $\Psi$:

1 $$f = \Psi s$$

$\Psi$ is a sparse orthogonal base of size $N \times N$, and *s* is a sparse coefficient. If the coefficient *s* has $k < < N$ non-zero coefficients, then $\Psi$ is said to be the sparse basis of the signal *f*.

The sampling process is a linear projection of the signal *f*:

2 $$y = \Phi f = \Phi \Psi s = As$$

where $\Phi$ is a projection matrix of size $m \times N$. *y* is a linear measure of size $m \times 1(m < N)$. In addition, the sensing matrix *A* should satisfy the RIP criteria^41^:

3 $$(1 - \delta_{k} )||x||_{2}^{2} \le ||Ax||_{2}^{2} \le (1 + \delta_{k} )||x||_{2}^{2}$$

wherein the equidistance constant $\delta_{k} \in (0,1)$, *k* is the number of coefficients *s* that are not zero.

The signal *f* measured using $\Phi$ can be recovered from *y*.

4 $$\min \left\| s \right\|_{0} s.t.\;y = As$$

In order to solve the above non-convex problems, many reconstruction algorithms have been proposed, such as orthogonal tracking algorithm (OMP)^42^, smooth norm ($SL_{0}$)^43^ and so on.

### Fractional Fourier transform

In order to improve the security of the system, the Fourier transform is improved to a fractional Fourier transform, and the required angle is used as a key to increase the key space and key sensitivity. First, the mathematical definition of one-dimensional fractional Fourier transform is introduced^44^:

5 $$F^{p} \{ f(x)\} (u) = \int_{ - \infty }^{ + \infty } {k_{p} } (x,u)f(x)dx$$

where the kernel function $k_{p} (x,u)$,

$$k_{p} (x,u) = \left\{ {\begin{array}{*{20}l} {A_{p} \;\exp [i\pi (u^{2} \cot \theta - 2ux\csc \theta + x^{2} \cot \theta )],} \hfill & {p \ne 2n} \hfill \\ {\delta (x + u),} \hfill & {p = 4n + 2} \hfill \\ {\delta (x - u),} \hfill & {p = 4n} \hfill \\ \end{array} } \right.$$

$$A_{p} = \sqrt {1 - i\cot \theta } ,\;\theta = p\pi /2,\;p \ne 2n$$

where $F^{p} \{ f(x)\} ( \cdot )$ is the *p*-order Fractional Fourier transform of signal $f(x)$, and *p* is the fractional order of Fractional Fourier transform. *x* and *u* respectively represent the input domain coordinates and the *p*-order fractional domain coordinates. $\theta$ represents the rotation angle of the time–frequency plane, $\delta ( \cdot )$ represents the impulse function, and $n$ is a positive integer.

The two-dimensional fractional Fourier transform is a generalization of the one-dimensional fractional Fourier transform. In the field of optics, a two-dimensional fractional Fourier transform is realized by optical instruments, which is defined as follows^45^:

6 $$F^{p1,p2} (u,v) = \int_{ - \infty }^{ + \infty } {\int_{ - \infty }^{ + \infty } {f(x,y)} } k_{p1,p2} (x,y,u,v)dxdy$$

$$f(x,y)$$ is the original signal, $$k_{p1,p2} (x,y,u,v)$$ is a kernel function, $$k_{p1,p2} (x,y,u,v) = k_{p1} (x,u) \times k_{p2} (y,v) = \tfrac{{\sqrt {(1 - i\cot \alpha )} \sqrt {(1 - i\cot \beta )} }}{2\pi }\exp [\frac{{i(x^{2} + u^{2} )}}{2\tan \alpha } - \frac{ixu}{{\sin \alpha }}]\exp [\frac{{i(y^{2} + v^{2} )}}{2\tan \beta } - \frac{iyv}{{\sin \beta }}]$$$$p1,p2$$ is expressed as a transformation order in the $$x,y$$ directions, and $$\alpha ,{\kern 1pt} \beta$$ represents a rotation angle.

### Chebyshev chaotic map

Since chaotic systems can generate pseudo-random sequences and are sensitive to initial values. In order to construct the sensing matrix, the Chebyshev chaotic system is used in this paper. The mathematical definition is as follows^46^:

7 $$r_{i + 1} = \tau (r_{j} ) = \cos (\alpha \cdot \arccos (r_{j} ))$$

$\alpha$ is a positive integer, $r_{j} \in [ - 1,1]$, when $r_{0} \in [ - 1,1]$ is the initial value, $R_{1} = \{ r_{j} = \tau^{j} (r_{0} )\} ,j = 0,1,2,...,r_{0}$ is a chaotic sequence. $\alpha ,r_{0}$ as the key of the cryptosystem. Chebyshev chaotic sequences are used to construct sensing matrices and random masks. That is to say, in the secure channel, we transmit the key instead of the perceptual matrix and the random mask. That is, the chaotic system controls the entire process.

## Image encryption and decryption process

Algorithm 1

1. The Chebyshev chaotic system generates a chaotic sequence $R_{{1}}$, and records the position sequence $Y$ corresponding to the sequence $R_{{1}}$.
2. The sequence $R_{{1}}$ is sorted in ascending order, and the corresponding position sequence $Y$ becomes chaotic sequence $Y_{1}$ as the order changes.
3. Select the first *m* numbers of the chaotic sequence $Y_{1}$ to get a subset.
4. Select *m* rows of matrix according to subset.

**Figure 1:**
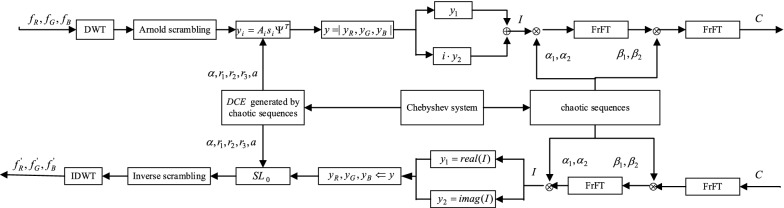
The proposed color image encryption and decryption scheme.

Figure 1 is the process of color image encryption and decryption based on compressed sensing and two-dimensional fractional Fourier transform.

### Encryption process

First, the Discrete Walsh transform (DWT) standard orthogonal basis sparse representation is performed on the three components of the color image from two directions:

8 $$f_{i} = \Psi s_{i} \Psi^{T} ,i = R,G,B$$

The three components of the color image are processed separately, and no other formats need to be converted.

The sparse signal is then measured using a chaotic sequence constructed by Chebyshev to construct a measurement matrix $\Phi$. The measurement matrix is defined as follows:

9 $$\Phi = \sqrt {\frac{N}{M}} DCE$$

Among them, *N* is the width of the image, $M = CR \times N$. The coefficient $\sqrt {\frac{N}{M}}$ is normalized to $DCE$ such that the energy of the measured value is close to the energy of the original signal. *D* is a random sampling operator, which is a random sample of the *m* rows of $CE$ according to a subset $\{1,2,\ldots,n\}$. In this paper, a chaotic system-based permutation algorithm is proposed for sampling. The essence of the permutation algorithm is to randomly select *m* rows using pseudo-random sequences generated by chaotic systems (algorithm 1).

$C$ is a cyclic matrix based on chaotic sequences. The size of *C* is $N \times N$, defined as follows.

10 $$C = \frac{1}{\sqrt n \upsilon (r)}\left( {\left. {\begin{array}{*{20}c} {r_{(n - 1)} } & {r_{(n - 2)} } & \cdots & {r_{0} } \\ {r_{0} } & {r_{(n - 1)} } & \cdots & {r_{1} } \\ \vdots & \vdots & \ddots & \vdots \\ {r_{(n - 2)} } & {r_{(n - 3)} } & \cdots & {r_{(n - 1)} } \\ \end{array} } \right)} \right.$$

where $r_{(i - 1)}$ is the *i*th element of the chaotic sequence $R_{1}$, $\upsilon (r)$ is the variance of the matrix $C$, $\frac{1}{\sqrt n \upsilon (r)}$ is for normalizing $C$, $C$ is for passing important information in $f$ to the measured value, and chaos-based cyclic matrix $C$ is only required for $n$ Element storage, which reduces memory requirements.

$E$ is a diagonal matrix in which diagonal elements are determined by chaotic sequences.

11 $$E_{i.i} = \left\{ \begin{gathered} + 1,\;0 \le r_{(i)} \le 1 \hfill \\ - 1,\; - 1 \le r_{(i)} \le 0 \hfill \\ \end{gathered} \right.\;i = 1,2,....,N$$

$r_{(i)}$ is the $i$ element of the sequence $R_{1}$. According to the nature of the Chebyshev sequence, the probability that the diagonal element $E_{i,i}$ in $E$ is equal to 1 or − 1 is the same. So $E$ is equivalent to a pseudo-randomizer that can change the sign of the signal.

Since the sampling subset $D$, the diagonal matrix $E$, and the chaotic sequence circulant matrix $C$ are all generated by the Chebyshev chaotic map, the $\Phi$ is a certain measurement matrix. To generate different measurement matrices, only the initial conditions of the Chebyshev system need to be changed.

Compressed sensing process is as follows:

12 $$y = \Phi \Psi s\Psi^{T} = As\Psi^{T}$$

The reconstruction algorithm (OMP or $SL_{0}$) can be used to recover $\Phi$, and finally the original signal is obtained by performing the inverse operation of the sparse coefficient and the sparse basis.

Finally, the measured image is subjected to two-dimensional fractional Fourier transform encryption using two random phase masks, which are generated based on the chaotic sequence. If the fractional Fourier transform is used directly, the data will explode, so CS has a major role in overcoming this defect.

The detailed encryption operations are as follows:

Step 1: The color image can be divided into three images according to the RGB component, respectively denoted as $f_{R} ,f_{G} ,f_{B} \in R^{N \times N}$. They are respectively sparsed by the sparse base $\Psi$ in the wavelet transform domain to obtain $f_{{1}} ,f_{{2}} ,f_{{3}}$. Then perform Arnold scrambling on $f_{{1}} ,f_{{2}} ,f_{{3}}$ to get $f^{\prime}_{{1}} ,f^{\prime}_{{2}} ,f^{\prime}_{{3}}$. Set the threshold TS, modify the elements of $f^{\prime}_{{1}} ,f^{\prime}_{{2}} ,f^{\prime}_{{3}}$, if the absolute value of the element is less than TS, change the element value to 0, get $f^{\prime\prime}_{1} ,f^{\prime\prime}_{2} ,f^{\prime\prime}_{3}$.

Step 2: Generating measurement matrix $\Phi$, the specific process is as follows:

- Given $\alpha_{1} ,r_{1} ,r_{2} ,r_{3}$ as the initial condition, the Chebyshev chaotic map is taken. The chaotic sequence $R_{i} = \{ r_{0} ,r_{1} ,...,r_{N - 1} \} ,i = 1,2,3$ is generated, and the matrix $C_{i} \in R^{N \times N}$ is obtained according to Eq. (10) by $R_{i}$.
- Obtain the matrix $E_{i} \in R^{N \times N}$ according to Eq. (11), obtain the sampling subset $D_{i}$ according to the algorithm 1, and finally obtain the $\Phi_{i}$ according to Eq. (9).

Step 3: The measurement matrix is measured in the three (stained) images of Eq. (8), which is compressed sensing. The measurement matrix $\Phi_{i}$ measures the three thinned images in Eq. (8), that is, the compressed sensing. The three measured values are obtained as $y_{R} ,y_{G} ,y_{B} \in R^{m \times N}$.

Step 4: Next, the two measured images are subjected to two-dimensional fractional Fourier transform encryption.

- Take the three measured images as an image $F$, the size is $m \times 3N$, divide $F$ into two parts from the middle, the left part is $y_{1}$, the right part is $y_{2}$, their size is $m \times \frac{3}{2}N$, and the two parts are combined into a complex number, $y_{1}$ is the real part, $y_{2}$ is the imaginary part.
  13 $$I(x,y) = y_{1} (x,y) + y_{2} (x,y)i$$
  $I(x,y)$ is a complex image.
- According to Eqs. (14)–(18), $as,r_{11} ,r_{22} ,r_{33}$ is calculated as the initial value to iterate the chaotic system $L + m \times N$ times, and the previous *L* times are discarded to obtain the chaotic sequence $L_{i} ,i = 1,2,3$.
  14 $$a = {{sum(f(:))} \mathord{\left/ {\vphantom {{sum(f(:))} {(N \times N \times 255)}}} \right. \kern-\nulldelimiterspace} {(N \times N \times 255)}}$$
  15 $$as = a - floor(a)$$
  16 $$r_{11} = \bmod ((as + r_{i} ),1)$$
  17 $$r_{22} = \bmod ((as + r_{2} ),1)$$
  18 $$r_{33} = \bmod ((as + r_{3} ),1)$$
  19 $$L_{i} = \{ l_{0} ,l_{1} ,...,l_{{m \times \frac{3}{2}N - 1}} \} ,i = 1,2,3$$
- $L_{1} ,L_{2}$ is used as a random phase mask for fractional Fourier transform, and the image is encrypted as:
  20 $$C(x,y) = FrFT^{{\alpha_{1} ,\alpha_{2} }} \{ FrFT^{{\beta_{1} ,\beta_{2} }} \{ I(x,y)\exp [iL_{1} (x,y)]\} \exp [iL_{2} (x,y)]\}$$

$L_{1} (x,y),L_{2} (x,y)$ is a two-phase random mask, and $\alpha_{{1}} ,\alpha_{2} ,\beta_{1} ,\beta_{2} \in [ - 2,2]$ is a fractional order in the $x,y$ direction, respectively.

Step 5: Perform global scrambling, ascending $L_{{3}}$, record the changed position $w$, $w$ as the address code to reorder the image $C$ to achieve scrambling effect. Convert the image $C$ into a one-dimensional matrix in the order of the columns, and scramble the one-dimensional matrix according to the following rules.

21 $$C_{1} (i) = C(w(i)),i = 1,2,...,m \times \frac{3}{2}N$$

Then, the scrambled matrix is converted into a two-dimensional matrix, and after being scrambled, the ciphertext is finally output as $C_{2}$.

### Decryption process

The decryption step is the inverse process of encryption.

Step 1: The anti-scrambling process, imitating step 5 of the encryption process, generates a chaotic sequence $L_{i} ,i = 1,2,3$ according to the key $as,r_{11} ,r_{22} ,r_{33}$, sorts $L_{{3}}$ to obtain an address code $w$, converts $C_{2}$ into a one-dimensional matrix $C_{{1}}$, and the assignment direction becomes:

22 $$C(w(i)) = C_{1} (i),i = 1,2,...,m \times \frac{3}{2}N$$

Convert to two-dimensional matrix $C$.

Step 2: Decrypt out $I(x,y)$:

23 $$I(x,y) = FrFT^{{ - \alpha_{1} , - \alpha_{2} }} \{ FrFT^{{ - \beta_{1} , - \beta_{2} }} \{ C(x,y)\exp [ - iL_{1} (x,y)]\} \exp [ - iL_{2} (x,y)]\}$$

Calculate the resulting complex-valued image and get two parts,

24 $$\left\{ \begin{gathered} y_{1} (x,y) = real\{ I(x,y)\} \hfill \\ y_{2} (x,y) = imag\{ I(x,y)\} \hfill \\ \end{gathered} \right.$$

Step 3: Think of $y_{1} ,y_{2}$ as an image, then divide it into three images, use $SL_{0}$ algorithm to reconstruct the image, Arnold inverse scrambling and then perform wavelet inverse transform to obtain $f_{R} ,f_{G} ,f_{B} \in R^{N \times N}$. Finally, the decrypted color image $f$ is obtained.

## Simulation results and performance analysis

### Simulation result

**Figure 2:**
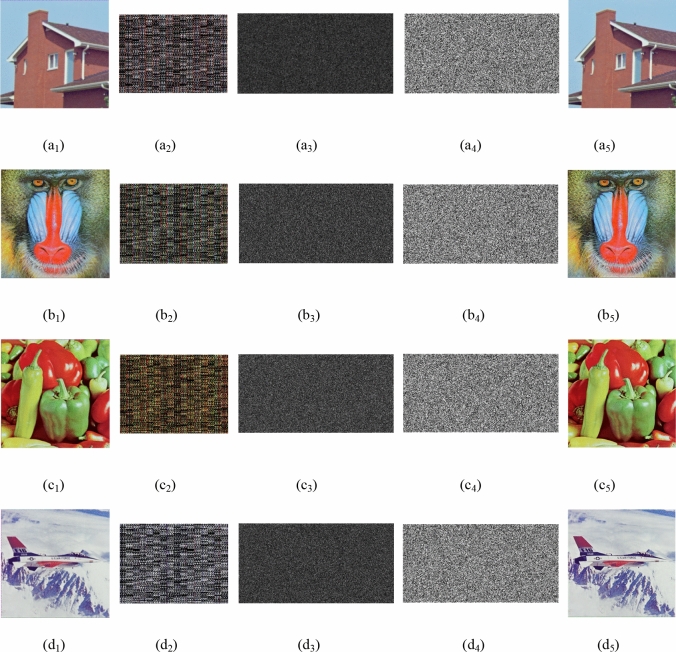
Encrypting and decrypting images: (**a**_**1**_)–(**d**_**1**_) are the original images “House”, “Baboon”, “Pepper” and “Airplane”, (**a**_**2**_)–(**d**_**2**_) are the results of 2D CS, (**a**_**3**_)–(**d**_**3**_) is the amplitude of the encrypted image, (**a**_**4**_)–(**d**_**4**_) is the phase of the encrypted image, (**a**_**5**_)–(**d**_**5**_) is the decrypted image.

In order to verify the feasibility and effectiveness of the encryption scheme, the security performance tests in this paper include key space, key sensitivity, correlation analysis, histogram analysis and various common attack tests. As shown in Fig. 2, matlab simulation experiments were performed using “House”, “Baboon”, “Pepper” and “Airplane” color images of size $256 \times 256 \times 3$, the corresponding TS are 10, 20, 10, 10. Figure 2a1–d1 are original images, Fig. 2a2–d2 are results of 2D CS, and Fig. 2a3–d3 are amplitudes of the encrypted image, the size of which is ${170} \times {384}$. The compression ratio is 0.664. Figure 2a4–d4 are the phases of the encrypted image, and Fig. 2a5–d5 are the decrypted images.

### PSNR analysis

Restoring an image includes decoding and reconstruction, using FrFT to decode under the correct key, and solving the l1 norm minimized reconstructed image is only similar to the plaintext image, so the quality of the decrypted image is evaluated using PSNR, and the formula is as follows:

25 $$PSNR = 10\log_{10} (\frac{255 \times 255}{{MSE}})$$

Of which,

26 $$MSE = \frac{1}{{N^{2} }}\sum\limits_{i = 1}^{N} {\sum\limits_{j = 1}^{N} {(f(i,j) - \tilde{f}(i,j))^{2} } }$$

**Figure 3:**
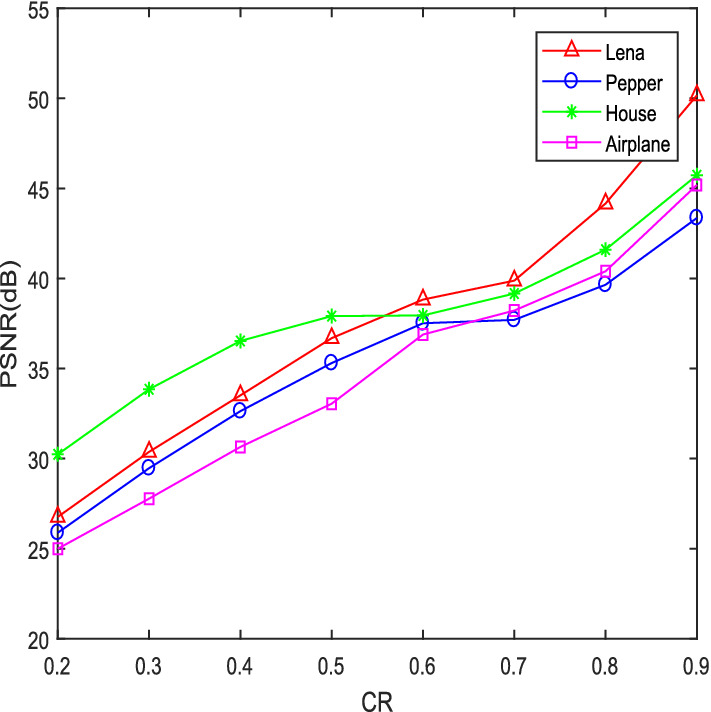
PSNR vs CR for different plain images.

$f(i,j),\;\tilde{f}(i,j)$ denotes the original image and the decrypted image respectively. Under the correct key, the decrypted image is as shown in Fig. 2. The PSNR of the five images is 38.8780, 37.9466, 28.5954, 37.6960, 38.0903, respectively. Therefore, the image decrypted by this algorithm is good. Figure 3 shows the PSNR values of different CRs of Lena, Pepper, House and Airplane images. The larger the CR, the larger the PSNR value, and the better the reconstruction effect. Table 1 shows the reconstruction effect of Pepper image of different CR. It can be seen from Table 1 that the compression performance of this algorithm is good. Taking the Lena as an example, the Table 2 lists the reconstruction performance comparison between this algorithm and other algorithms. With the same CR from the Table 2, the reconstruction quality of this algorithm is better.

**Table 1:**
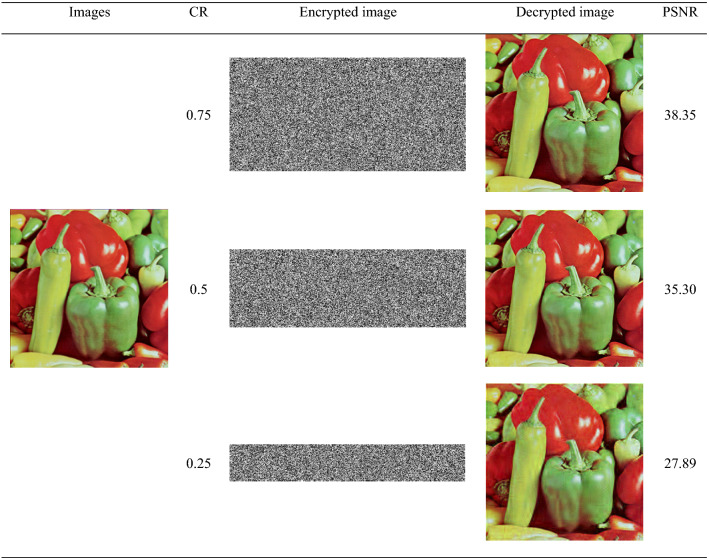
PSNR values for different compression ratios.

**Table 2:** The compression performance of different algorithms.

Plain image	CR	Our	Ref.^22^	Ref.^25^	Ref.^38^
Lena	0.25	28.74	26.52	26.06	–
	0.5	36.68	29.23	29.82	> 25
	0.75	41.94	29.21	29.56	> 29

### Histogram analysis

**Figure 4:**
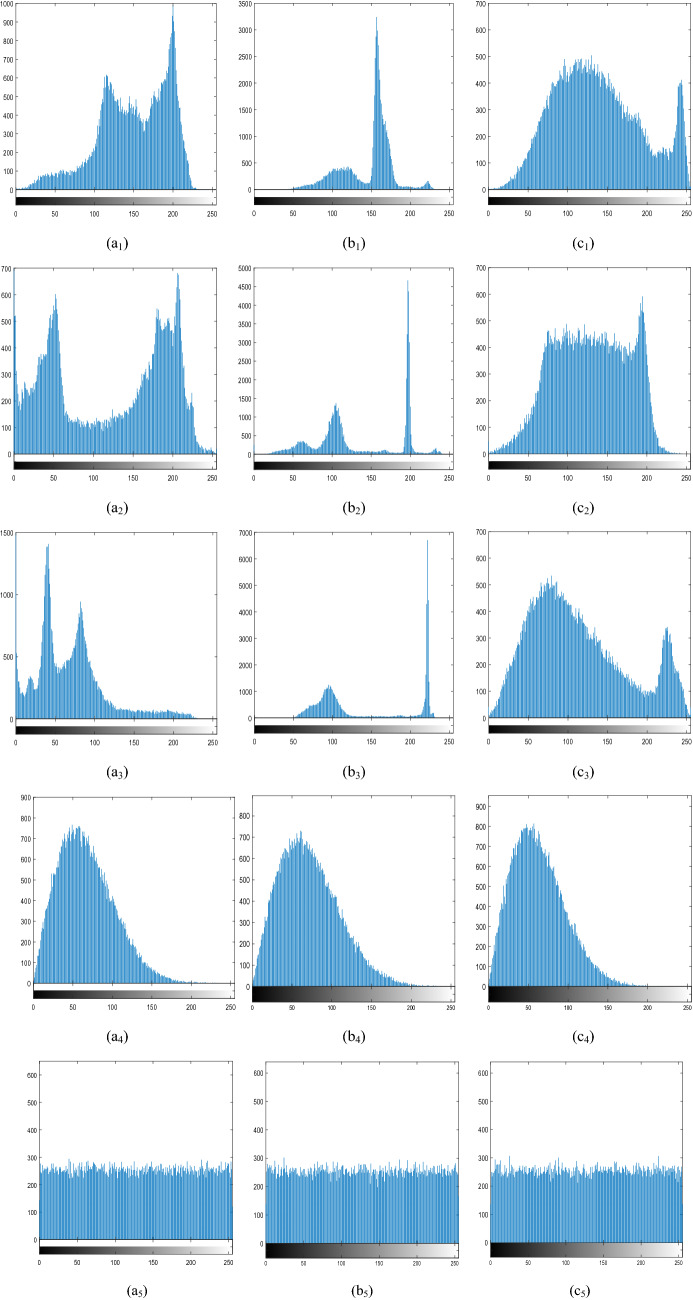
The histograms of plaintexts and ciphertexts: The original images “Pepper”, “House” and “Baboon”. (**a**_**1**_)–(**c**_**1**_) The histogram of original images R component, (**a**_**2**_)–(**c**_**2**_) The histogram of original images G component, (**a**_**3**_)–(**c**_**3**_) The histogram of original images B component, (**a**_**4**_)–(**c**_**4**_) The histogram of amplitude of encrypted images, (**a**_**5**_)–(**c**_**5**_) The histogram of phase of encrypted images.

Histogram analysis of important indicators of image security after image encryption^47^. As shown in Fig. 4a1–a3, b1–b3, c1–c3 represents the R, G, and B components of the three color images of “Lena”, “House” and “Baboon”, respectively, a_4_–c_4_, a_5_–c_5_ respectively represent the amplitude and phase after the three images are encrypted. Obviously, the histograms of the R, G, and B components of the three original images are different from each other, but different images are encrypted with very similar histograms, that is, the attacker cannot obtain useful messages from the ciphertext histogram.

### Adjacent pixel correlation

**Figure 5:**
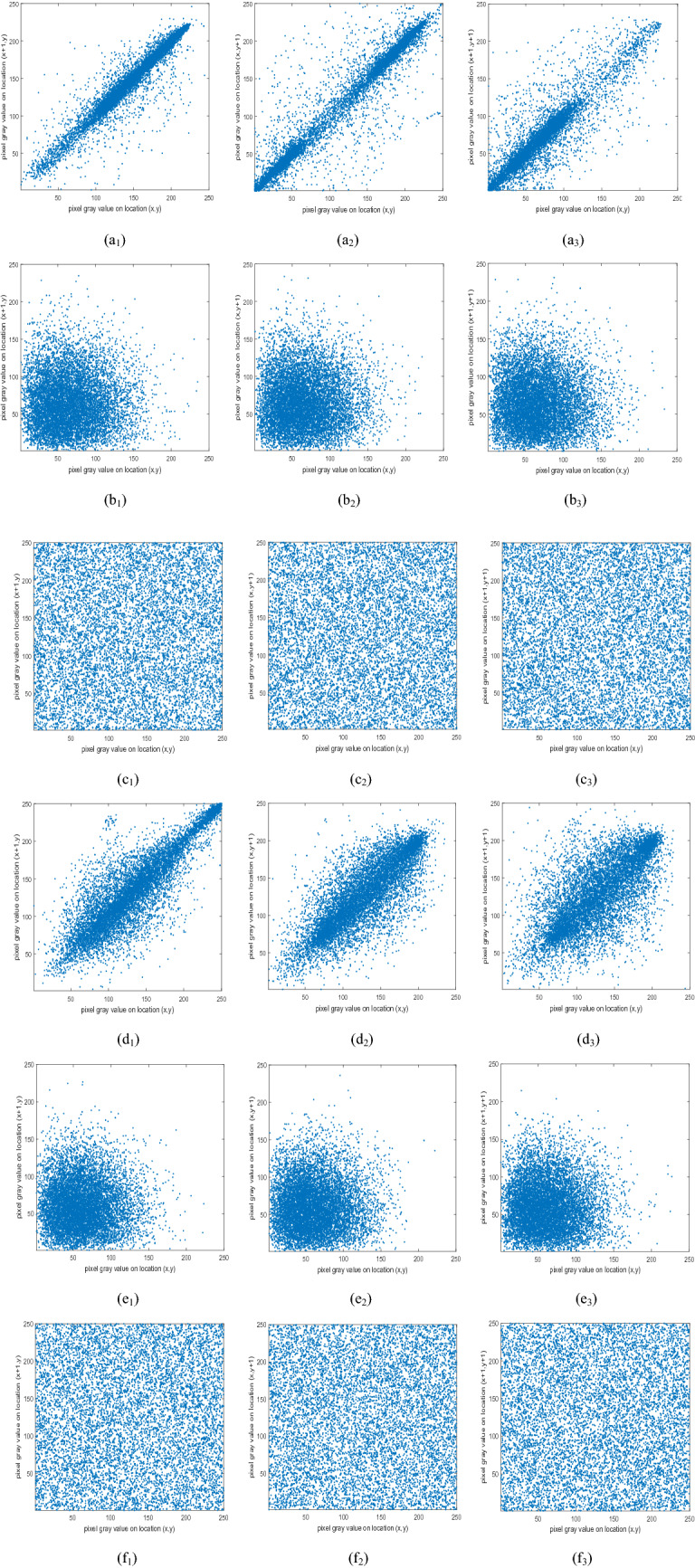
Horizontal, vertical, diagonal correlation test results for “Pepper” and “Baboon” plaintext images and their ciphertext images: (**a**_**1**_)–(**a**_**3**_), (**d**_**1**_)–(**d**_**3**_) represent the horizontal, vertical and diagonal correlation distribution of the plaintext image, (**b**_**1**_)–(**b**_**3**_), (**c**_**1**_)–(**c**_**3**_), (**e**_**1**_)–(**e**_**3**_), (**f**_**1**_)–(**f**_**3**_) represent the horizontal, vertical and diagonal correlation distribution of the amplitude and phase of the ciphertext, respectively.

Randomly select the plaintext images R, G, B three channels and the amplitude and phase of the ciphertext on 2000 pairs of pixels for correlation testing^48^. The simulation results are shown in Fig. 5, from a_1_–a_3_, d_1_–d_3_, it is found that the correlation of the plaintext images in the horizontal, vertical, and diagonal directions is concentrated, showing a clear linear relationship, from b_1_–b_3_, c_1_–c_3_, e_1_–a_3_, f_1_–f_3_ found that the encrypted image pixel values are evenly distributed and scattered, indicating that the algorithm proposed in this paper makes the statistical features of the plaintext image spread evenly into the ciphertext.

Randomly select the plaintext images R, G, B three channels and the amplitude and phase of the ciphertext on 2000 pairs of pixels for correlation testing. According to Eq. (27), the correlation coefficient of horizontal, vertical and diagonal angles is measured, and the operation is repeated 100 times to calculate the average of the correlation coefficients of horizontal, vertical and diagonal. The final statistical results are shown in Table 3.

27 $$r_{xy} = \frac{{{\text{cov}} (x,y)}}{{\sqrt {D(x)} \sqrt {D(y)} }}$$

Of which,

$${\text{cov}} (x,y) = \frac{1}{N}\sum\limits_{i = 1}^{N} {(x_{i} - E(x))(y_{i} - E(y))} ,\quad D(x) = \frac{1}{N}\sum\limits_{i + 1}^{N} {(x_{i} - E(x))^{2} } ,\quad E(x) = \frac{1}{N}\sum\limits_{i = 1}^{N} {x_{i} } .$$

It can be seen from Table 3 that the correlation coefficients of the plain image in the horizontal, vertical, and diagonal adjacent pixels are large. After encryption, the correlation coefficients of the ciphertext in the horizontal, vertical, and diagonal adjacent pixels are small and both at 1%. The algorithm proposed in this paper can effectively reduce the correlation of adjacent pixels. It can be seen from Table 4 that the encrypted correlation coefficient of this paper is lower than that most algorithms, so the encryption scheme of this paper can resist statistical analysis.

**Table 3:** The correlation coefficient of adjacent pixels.

Correlation coefficient	The original images			Our encrypted images	
	R	G	B	Amplitude	Phase
**Lena**					
Horizontal	0.9389	0.9392	0.8932	0.0027	− 0.0000
Vertical	0.9677	0.9688	0.9380	− 0.0027	− 0.0032
Diagonal	0.9090	0.9114	0.8474	0.0003	0.0026
**House**					
Horizontal	0.9670	0.9800	0.9818	− 0.0013	− 0.0055
Vertical	0.9353	0.9718	0.9747	0.0033	− 0.0014
Diagonal	0.9127	0.9561	0.9621	0.0004	− 0.0059
**Baboon**					
Horizontal	0.9135	0.8027	0.8774	0.0002	− 0.0045
Vertical	0.8743	0.7570	0.8651	− 0.0001	0.0065
Diagonal	0.8530	0.7010	0.8161	0.0027	− 0.0007
**Pepper**					
Horizontal	0.9526	0.9620	0.9418	0.0035	0.0004
Vertical	0.9554	0.9688	0.9545	− 0.0054	0.0023
Diagonal	0.9179	0.9365	0.9097	0.0012	0.0020
**Airplane**					
Horizontal	0.9107	0.9103	0.9257	0.0021	− 0.0008
Vertical	0.8947	0.9048	0.8728	0.0015	− 0.0042
Diagonal	0.8292	0.8464	0.8364	− 0.0033	− 0.0030

**Table 4:** Comparison of this algorithm with other algorithms.

Correlation coefficient	Encrypted images	
	Amplitude	Phase
**Lena**		
Horizontal	0.0027	− 0.0000
Vertical	− 0.0027	− 0.0032
Diagonal	0.0003	0.0026
**Ref.**^9^		
Horizontal	–	0.0026
Vertical	–	− 0.0038
Diagonal	–	0.0062
**Ref.**^10^		
Horizontal	–	0.0001
Vertical	–	0.0089
Diagonal	–	0.0091
**Ref.**^11^		
Horizontal	–	0.0044
Vertical	–	0.0151
Diagonal	–	0.0012
**Ref.**^36^		
Horizontal	0.0127	0.0127
Vertical	0.0101	− 0.0271
Diagonal	0.0139	0.0183
**Ref.**^37^		
Horizontal	0.0104	0.0158
Vertical	0.0299	0.0158
Diagonal	0.0062	− 0.0339
**Ref.**^38^		
Horizontal	0.2905	− 0.0117
Vertical	0.4711	− 0.2089
Diagonal	0.2894	0.0301

### Information entropy

Test image randomness using entropy. If the entropy value is closer to 8, it means that the pixels of the image are more uniform. The formula for calculating entropy is as follows:

28 $$H(g) = \sum\limits_{i = 0}^{{2^{L} - 1}} {P(g_{i} )} \log_{2} \frac{1}{{P(g_{i} )}}$$

where *g* represents a set of pixels. $P(g_{i} )$ is the probability of occurrence of *g*, and *L* is the total number of $g_{i}$. Table 5 shows the entropy corresponding to different images and comparison with other algorithms. The table shows that the encrypted image is close to 8, which means that it is safe against entropy attacks. Moreover, our algorithm is larger than the entropy value of the literature^9,10,12^, which shows that our algorithm is effective.

**Table 5:** Information entropy of different images.

Algorithm	R	G	B	Cipher
Lena	7.2775	7.5869	7.0133	7.9959
House	7.6756	7.4794	7.7526	7.9962
Baboon	6.4311	6.5389	6.2320	7.9959
Pepper	7.3449	7.5718	7.1005	7.9959
Airplane	6.8567	6.9602	6.3352	7.9954
Lena in Ref.^9^		7.3441		7.9832
Lena in Ref.^10^		7.7583		7.9912
Lena in Ref.^12^		–		7.9896

### Key space analysis

When an attacker uses a violent attack, this requires enough key space to prevent the attacker from obtaining any information without the correct key^36^. In this algorithm, take lena picture as an example, the control parameters of the Chebyshev chaotic system are $\alpha = 8$, the initial value $r_{1} = 0.{091},r_{2} = 0.473,r_{1} = 0.782$ is related to the plaintext control parameter $a = {1}{\text{.5041015625}}$, the parameters of Arnold scrambling are $b_{1} = 8,b_{2} = 7,t = 80$, and the two-dimensional Discrete Fractional Fourier Transform (DFrFT) transform angle is $\alpha_{1} = 1.41,\alpha_{2} = 0.6,\beta_{1} = { - }0.41,\beta_{2} = { - }1.6$. Using Matlab for simulation experiments, the calculation accuracy is ${10}^{{ - 15}}$, The control parameters, initial conditions and Arnold scrambling parameter key space are all ${10}^{{{15}}}$, i.e. $S_{\alpha } = S_{{r_{1} }} = S_{{r_{2} }} = S_{{r_{3} }} = S_{a} = S_{{b_{1} }} = S_{{b_{2} }} = S_{t} = 10^{15}$; the transformation angle key space of the two-dimensional DFrFT is $S_{{\alpha_{1} }} = S_{{\alpha_{2} }} = S_{{\beta_{1} }} = S_{{\beta_{2} }} = 10^{6}$; By calculating, the system key space is $S = S_{\alpha } \cdot S_{{r_{1} }} \cdot S_{{r_{2} }} \cdot S_{{r_{3} }} \cdot S_{a} \cdot S_{{b_{1} }} \cdot S_{{b_{2} }} \cdot S_{t} \cdot S_{{\alpha _{1} }} \cdot S_{{\alpha _{2} }} \cdot S_{{\beta _{1} }} \cdot S_{{\beta _{2} }} = 10^{{144}}$. It is much larger than the key space of each algorithm in Table 6, so the algorithm can resist brute force attacks.

**Table 6:** Comparison of key space.

Algorithm	Proposed algorithm	Ref.^9^	Ref.^11^	Ref.^12^	Ref.^38^
Key space	$10^{144}$	$10^{90}$	$10^{90}$	$10^{45}$	$10^{91}$

### Key sensitivity analysis

**Figure 6:**
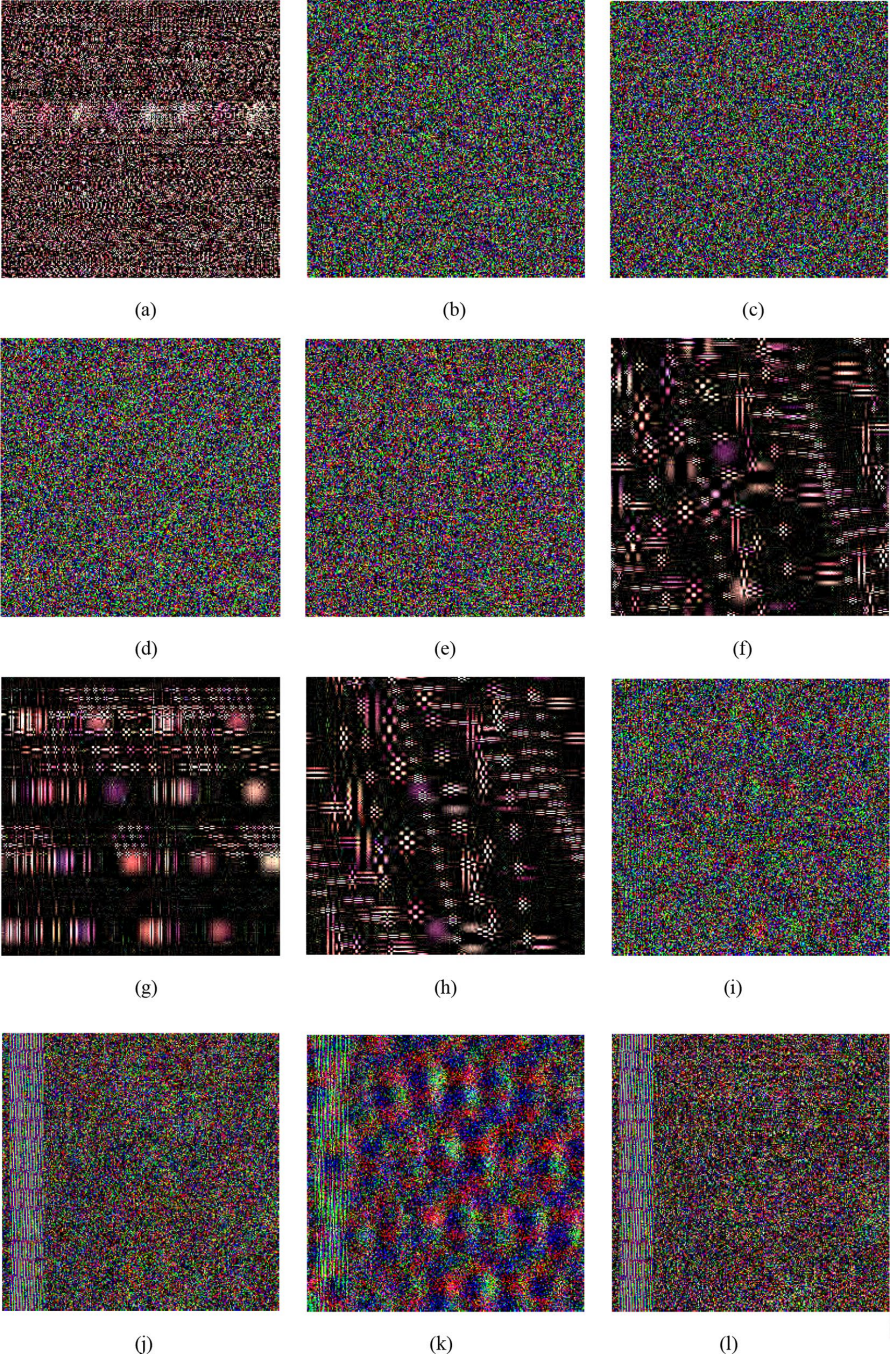
Decrypted “Lena” with incorrect (**a**) $\alpha$, (**b**) $a$, (**c**) $r_{1}$, (**d**) $r_{2}$, (**e**) $r_{3}$, (**f**) $b_{1}$, (**g**) $b_{{2}}$, (**h**) $t$, (**i**) $\alpha_{1}$, (**j**) $\alpha_{2}$, (**k**) $\beta_{1}$, (**l**) $\beta_{2}$.

The key sensitivity of the algorithm is very strong, any key small changes, other keys remain unchanged, under the correct encryption algorithm, cannot decrypt the correct plaintext image^49^. The simulation experiment results are shown in Fig. 6a–e respectively represent the decrypted image of $\alpha = {8} + {10}^{{ - 15}}$, $a = {1}{\text{.5041015625}} + {10}^{{ - 15}}$, $r_{1} = 0.{0}9{1} + 10^{ - 15}$,$r_{1} = 0.{473} + 10^{ - 15}$,$r_{1} = 0.{782} + 10^{ - 15}$. Figure 6f–h respectively represent the decrypted image of $b_{1} = 9$, $b_{2} = 8$, $t = 79$. Figure 6i–l respectively represent the decrypted image of $\alpha_{{1}} = {1}{\text{.41}} + {0}{\text{.01}}$,$\alpha_{{1}} = {0}{\text{.6}} + {0}{\text{.01}}$,$\beta_{{1}} = { - 0}{\text{.41}} + {0}{\text{.01}}$,$\beta_{{2}} = {1}{\text{.6}} + {0}{\text{.01}}$. Experiments have shown that small changes in the key have a great impact on decryption.

**Figure 7:**
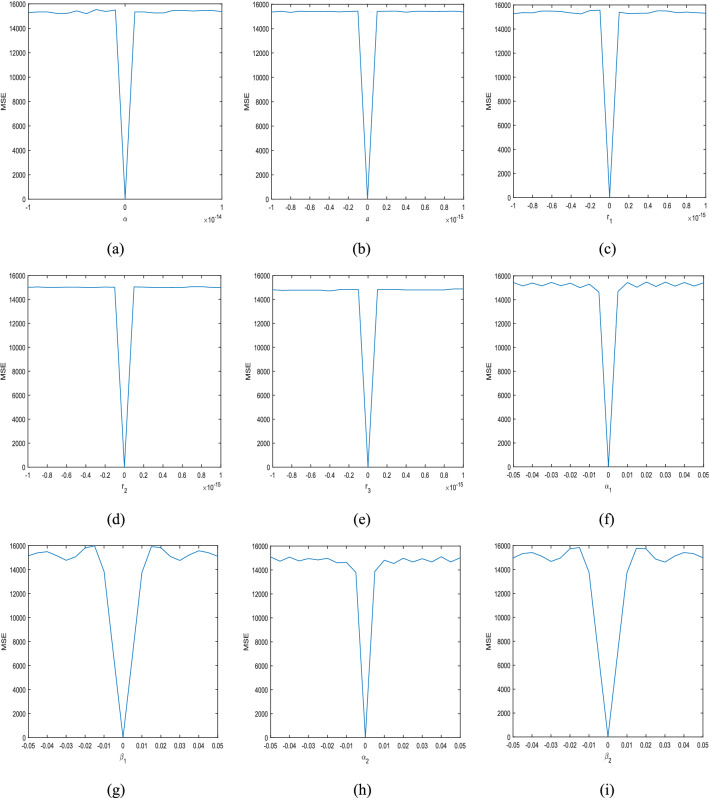
MSE curves for (**a**) $\alpha$, (**b**) $a$, (**c**) $r_{1}$, (**d**) $r_{2}$, (**e**) $r_{3}$, (**f**) $\alpha_{1}$, (**g**) $\alpha_{2}$, (**h**) $\beta_{1}$, (**i**) $\beta_{2}$.

Figure 7a shows the mean square error (MSE) curve of the deviation of the control parameter $\alpha$. Try different values in the range $[{ - }10^{{{ - }14}} ,10^{{{ - }14}} ]$, step size is $10^{{{ - }1{5}}}$. Figure 7b–e shows the MSE curve of the deviation of initial condition $a$, $r_{1}$, $r_{2}$, $r_{3}$. Try different values in the range $[ - 10^{ - 15} ,10^{ - 15} ]$, step size is $10^{{{ - }1{6}}}$. It can be seen that the key is slightly transformed, the MSE is large, and the original image cannot be seen in the decrypted image. Figure 7f–i show the MSE graph of the deviation of the FrFT order $\alpha_{{1}} ,\alpha_{{2}} ,\beta_{{1}} ,\beta_{{2}}$, and it can be seen that the order is slightly changed, and the MSE is large. Therefore, this algorithm is very sensitive to keys.

### Noise attack

**Figure 8:**
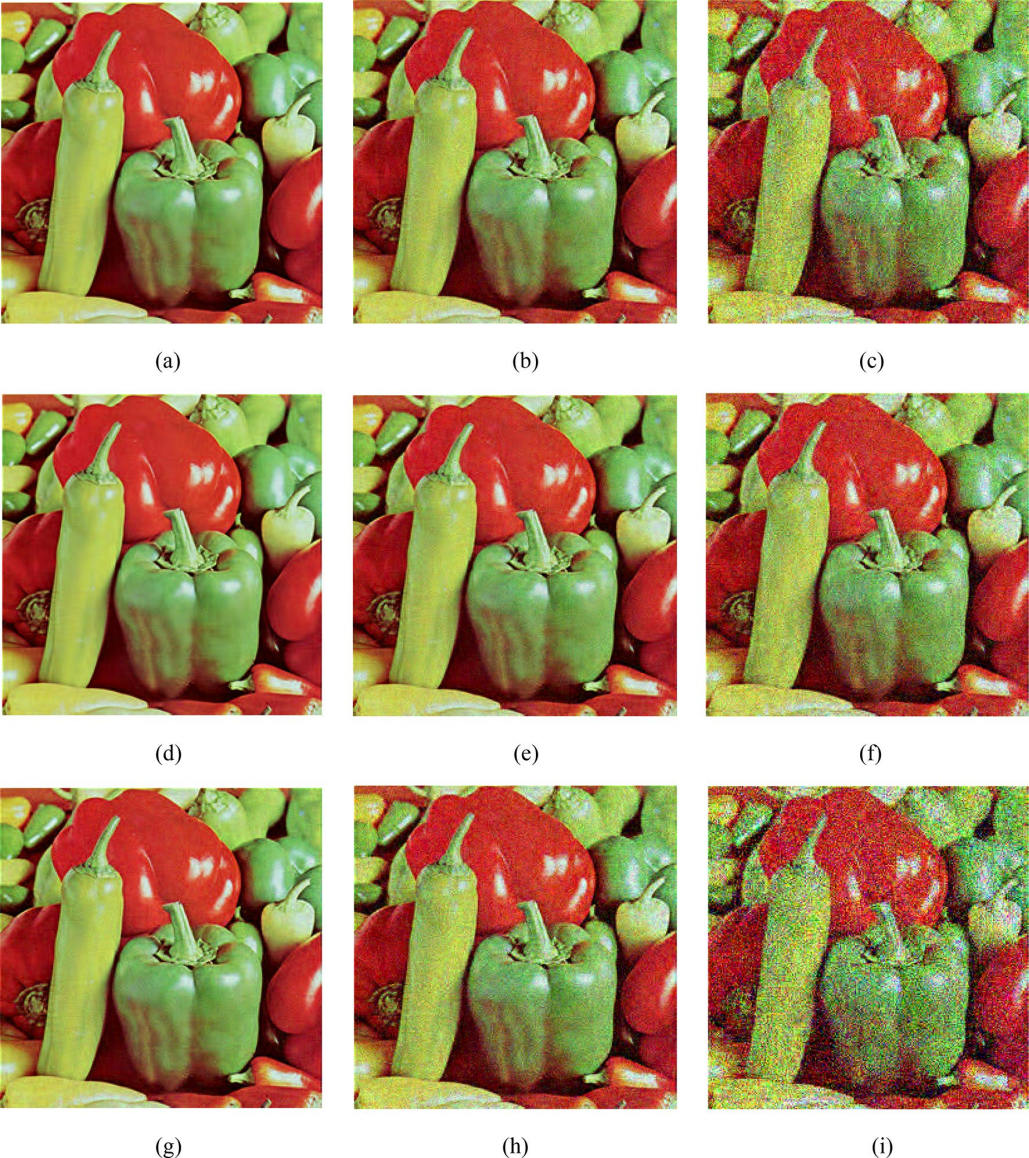
The results of noise attack with different noise strengths: (**a**) ${10}^{{ - 5}}$ GN, (**b**) ${10}^{{ - 4}}$ GN, (**c**) ${10}^{{ - 3}}$ GN, (**d**) ${10}^{{ - 4}}$ SPN, (**e**) ${10}^{{ - 3}}$ SPN, (**f**) ${10}^{{ - 2}}$ SPN, and (**g**) ${10}^{{ - 4}}$ SN, (**h**) ${10}^{{ - 3}}$ SN, (**i**) ${10}^{{ - 2}}$ SN.

Next, we test the resistance of this algorithm to noise. Take the Pepper as an example, add Gaussian noise (GN) with mean value of 0 and variance of ${10}^{{ - 5}}$, ${10}^{{ - 4}}$, ${10}^{{ - 3}}$ to the encrypted image, and the decrypted images are shown in Fig. 8a–c. Add Salt and Pepper noise (SPN) with intensity ${10}^{{ - 4}}$, ${10}^{{ - 3}}$, ${10}^{{ - 2}}$ to the encrypted image, and the decrypted images are shown in Fig. 8d–f. Add Speckle noise (SN) with intensity ${10}^{{ - 4}}$, ${10}^{{ - 3}}$, ${10}^{{ - 2}}$ to the encrypted image, and the decrypted images are shown in Fig. 8g–i. Decrypting the noise-added image can see the rough information of the original image, so this algorithm has better robustness. Table 7 compares the PSNR value of the decrypted image and the Lena plaintext image when the encrypted image is attacked by GN, SPN, and SN when the compression rate is 50% with the algorithm^25^. It can be seen from the table that this algorithm has a stronger ability to resist noise attack. Add random noise of different intensities to the Lena ciphertext image, as shown in Eq. (29). The Table 8 is the PSNR value of the decrypted image and the plaintext image with random noise added with different intensities. It can be seen that the quality of the restored image by this algorithm is relatively high under the same intensity.

29 $$I = I + k \times Noise$$

**Table 7:** The anti-noise performance comparison of two methods in the 50% sampling rate.

Image	GN			SPN			SN		
	$10^{ - 6}$	$3 \times 10^{ - 6}$	$5 \times 10^{ - 6}$	$10^{ - 6}$	$3 \times 10^{ - 6}$	$5 \times 10^{ - 6}$	$10^{ - 6}$	$3 \times 10^{ - 6}$	$5 \times 10^{ - 6}$
Proposed	34.8113	33.2051	32.4611	35.3472	35.3472	35.3472	35.3472	34.5733	33.9705
Ref.^25^	30	< 28	< 27	31	< 31	< 30	31	< 32	< 32

**Table 8:** The anti-noise performance comparison of two methods in the 75% sampling rate.

Image	Random noise attack k = 0.1	Random noise attack k = 0.3	Random noise attack k = 0.6
Proposed	41.3867	38.7516	35.1461
Ref.^39^	20.09	12.27	9.58

### Clipping attack

**Figure 9:**
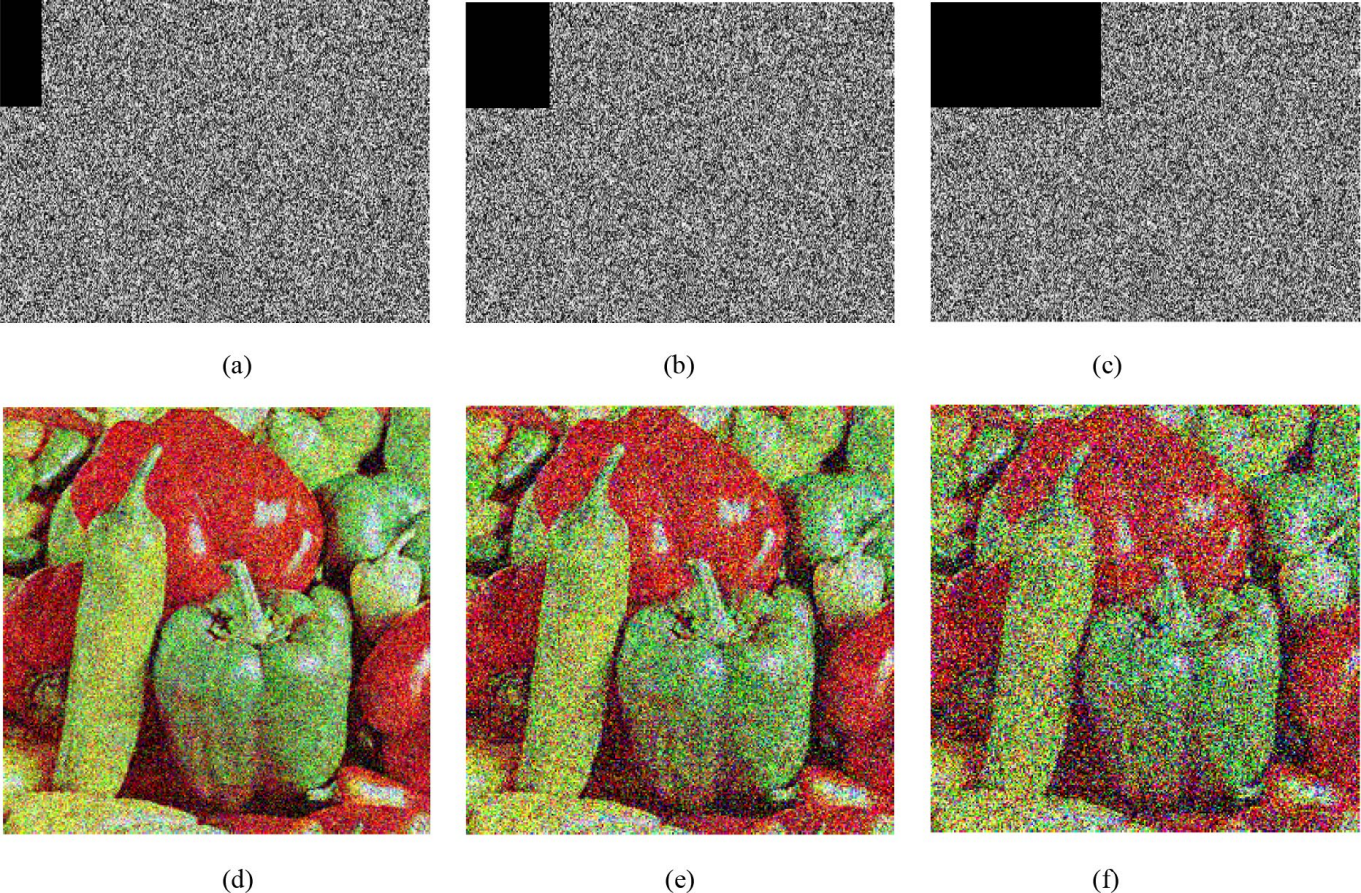
Robustness of the encryption scheme: (**a**) encrypted image with 5% data loss, (**b**) encrypted image with 10%data loss, (**c**) encrypted image with 20% data loss. (**d**)–(**f**) are corresponding decrypted images of (**a**)–(**c**), respectively.

When the ciphertext is subjected to a tailoring attack during transmission, there is no doubt that the quality of the decrypted image will decrease. Figure 9 shows three different clipping methods and their recovery results. Experiments show that although the decrypted image is a rough version of the original image, the main information of the original image can still be represented by the correct key. Experiments have shown that encryption algorithms can resist tailoring attacks. Table 9 is a comparison of the PSNR of Lena's decrypted image and plaintext image with other algorithms. The image is restored after 5%, 10%, and 20% loss of encrypted image data. It can be seen that the PSNR is lower as the data is lost more. Compared with the algorithm^39^, the PSNR value of this algorithm is dominant in the data loss of 5%, but with the increase of data loss, it is not dominant. Therefore, our algorithm can resist tailoring attacks to a certain extent.

**Table 9:** The comparison of performance against cropping of two algorithms in the 75% sampling rate.

Data loss intensity (%)	PSNR	Ref.^39^
5	13.25	12.51
10	10.21	10.67
20	7.19	8.72

### Differential attack

To test whether an encryption scheme is good, NPCR (Number of Pixels Change Rate) and the UACI (Unified Average Changing Intensity) are important standards. If a slight change is made to the plaintext pixel value, which corresponds to a large change in the encrypted pixel value, it means that the encryption scheme is good. NPCR and UACI are the numerical response of this standard. The calculation method of NPCR and UACI is as follows^11^:

30 $$NPCR = \frac{{\sum\nolimits_{i,j} {D(i,j)} }}{W \times H} \times 100\% ,$$

and

31 $$UACI = \frac{1}{W \times H}\left[ {\sum\limits_{i,j} {\frac{{|d_{1} (i,j) - d_{2} (i,j)|}}{255}} } \right] \times 100\% .$$

Here *M* and *N* respectively represent the width and height of the image, and $d_{1}$ and $d_{{2}}$ are the two ciphertext images after the original plaintext image has been changed by one pixel value. If $d_{{1}} (i,j) \ne d_{2} (i,j)$, then $D(i,j) = {1}$, otherwise, $D(i,j) = 0$. We add 1 to any pixel value, calculate 100 groups, and take the average to get Table 10. It can be seen from Table 10 that the NPCR obtained by the encryption scheme is about 99.60%, and the UACI is greater than 33%. Table 11 is the comparison result between this algorithm and other algorithms. We can find that although our results are not the best, they can resist differential attacks.

**Table 10:** The mean NPCR and UACI of ciphered images.

Images	NPCR (%)	UACI (%)
Lena	99.6078	33.4531
House	99.6323	33.4499
Baboon	99.6246	33.3373
Pepper	99.5818	33.3626
Airplane	99.6155	33.1816

**Table 11:** Comparison of NPCR and UACI on ‘Lena’.

Lena images	NPCR (%)	UACI (%)
Proposed algorithm	99.6078	33.4531
Ref.^9^	99.66	33.62
Ref.^10^	1	33.47
Ref.^11^	99.62	33.45
Ref.^12^	99.6090	33.4727
Ref.^13^	99.6155	33.2744
Ref.^14^	99.6017	28.1370

### The influence of different sparse and reconstruction methods on encryption and decryption results

**Figure 10:**
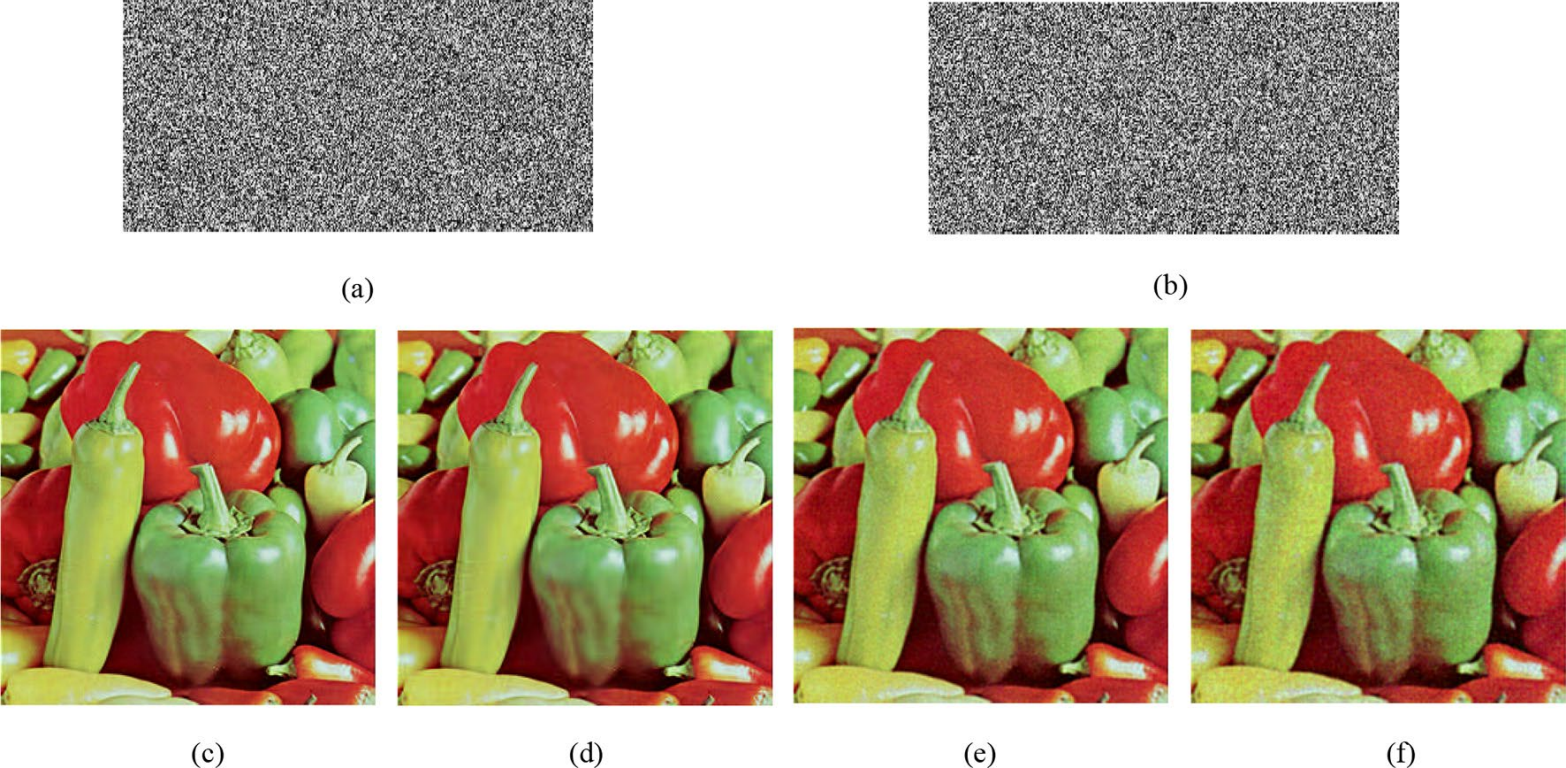
Simulation results of different sparse and reconstruction methods for Pepper.

**Figure 11:**
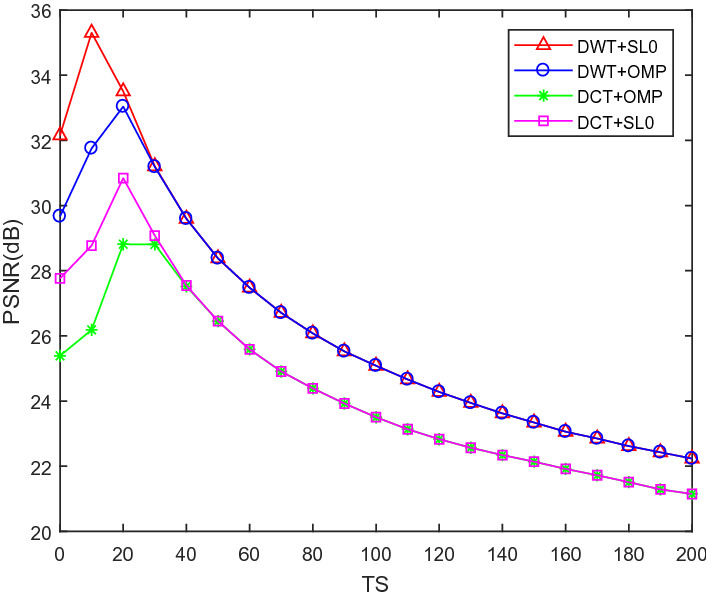
PSNR vs TS for Pepper with different sparse and reconstruction methods.

To analyze the impact of sparse methods and reconstruction methods, we use DWT and DCT sparse 256 $\times$ 256 Pepper, and use OMP and SL to reconstruct image. As shown in Fig. 10, (a) is an encrypted image using DWT, (b) is an encrypted image using DCT, (c) is an image reconstructed using DWT sparse and SL0, and (d) is an image reconstructed using DWT sparse and OMP, (e) is an image reconstructed using DCT sparse and SL0, (f) is an image reconstructed using DCT sparse and OMP. It can be seen from the figure that using DWT sparse, the reconstructed visual quality is better. Figure 11 shows the relationship between the reconstruction effect and the threshold TS. It can be seen that when TS = 10, using DWT sparse, the PSNR value of SL0 reconstruction is the largest.

### Time analysis

In practical applications, both safety performance and time must be considered. As shown in Tables 12 and 13, this paper analyzes the encryption and decryption time of different sizes of images and different CRs. It can be seen from the table that for the same image, different CRs have a slight impact on the time. For 256 $\times$ 256 images, the encryption time range is 1.5–2, and for 512 $\times$ 512 images, the encryption time range is 5–6. For 256 $\times$ 256 images, the decryption time range is 3–5, for 512 $\times$ 512 images, the decryption time range is 10–15. The reason for the increase in the decryption time is that the reconstruction process takes a long time to find the optimal solution. When CR is equal, as the image size increases, the encryption and decryption process takes more time. Therefore, in practice, CR and time are comprehensively considered for selection. Table 14 compares the time with other algorithms. As shown in the table, our algorithm takes the shortest time.

**Table 12:** Encryption time (second).

Images size	Lena 256 $\times$ 256	Baboon 256 $\times$ 256	Pepper 512 $\times$ 512	Airplane 512 $\times$ 512
CR = 0.25	1.67	1.65	5.13	5.15
CR = 0.5	1.80	1.72	5.79	5.51
CR = 0.75	1.79	1.81	6.35	6.04

**Table 13:** Decryption time (second).

Images size	Lena 256 $\times$ 256	Baboon 256 $\times$ 256	Pepper 512 $\times$ 512	Airplane 512 $\times$ 512
CR = 0.25	4.59	3.94	10.37	10.97
CR = 0.5	4.24	4.26	13.31	11.50
CR = 0.75	4.70	4.47	14.58	13.78

**Table 14:** The encryption time comparison results with other algorithms (second).

Images size	Lena 256 $\times$ 256	Baboon 256 $\times$ 256	Pepper 256 $\times$ 256
Proposed	1.67	1.65	1.79
Ref.^4^	3.23	3.53	3.68
Ref.^5^	11.12	11.45	12.13
Ref.^9^	2.25	2.55	2.76

## Conclusion

This paper combines the advantages of structured random perceptual matrix and chaos to obtain a structured sensing matrix measurement image. A compression-based and two-dimensional fractional Fourier image encryption is proposed. This paper first compresses and encrypts through CS, and then re-encrypts through 2D FrFT. The inverse scrambling matrix, the chaotic cyclic matrix, the sampling subset and the double random phase mask are generated by the Chebyshev chaotic sequence, that is, the chaotic system controls the encryption process. Simulation experiments show that the proposed algorithm has good resilience and robustness. It can not only resist statistical analysis, noise attack and tailoring attacks, but also has a large key space and is sensitive to keys. Therefore, the algorithm has good performance and security.
